# Development and Evaluation of a Deep Learning Model for Ovarian Cancer Histotype Classification Using Whole-Slide Imaging

**DOI:** 10.3390/jimaging12040144

**Published:** 2026-03-25

**Authors:** Dagoberto Pulido, Nathalia Arias-Mendoza

**Affiliations:** 1 Centro de Investigación en Ciencia Aplicada y Tecnología Avanzada, Unidad Querétaro (CICATA-Querétaro), Instituto Politécnico Nacional (IPN), Santiago de Querétaro 76090, Mexico; 2 Martinos Center for Biomedical Imaging, Massachusetts General Hospital, Harvard Medical School, Boston, MA 02114, USA; 3 Ingeniería en Desarrollo de Software, Universidad Virtual del Estado de Guanajuato (UVEG), Purísima de Bustos, Guanajuato 36400, Mexico

**Keywords:** ovarian carcinoma, interobserver variability, diagnostic support system, histopathological subtyping, computational pathology, multiple instance learning (MIL), attention mechanisms, whole-slide images (WSIs), deep learning, artificial intelligence

## Abstract

The histopathological classification of ovarian carcinoma is fundamental for patient management. While microscopic evaluation by pathologists is the current diagnostic standard, it is known to be subject to interobserver variability, which can affect consistency in treatment decisions. This study addresses this clinical need by developing and validating a deep learning-based diagnostic support tool designed to enhance the objectivity and reproducibility of this classification. In this work, we address a key challenge in computational pathology—the tendency of attention mechanisms to overfit by concentrating on limited features—by systematically evaluating a direct regularization method within multiple instance learning (MIL) models. The models were trained and validated using 10-fold cross-validation on a public training set of 538 whole-slide images and further tested on an independent public dataset for the more challenging task of molecular subtype classification. We utilized features from a foundational model pre-trained on histopathology data to represent tissue morphology. Our findings demonstrate that directly regularizing the attention mechanism with a stochastic approach provides a statistically significant improvement in accuracy and generalization, highlighting its power as a robust technique to mitigate overfitting for this clinical task. In direct contrast to the reported variability in manual assessment, our final model achieved high consistency and accuracy, with a balanced accuracy of 0.854 and a Cohen’s Kappa of 0.791. The model also demonstrated strong generalization on the molecular classification task. Its attention mechanism provides visual heatmaps for pathologist review, fostering interpretability and trust. We have developed a highly accurate and generalizable artificial intelligence tool that directly addresses the challenge of interobserver variability in ovarian cancer classification. Its performance highlights the potential for artificial intelligence to serve as a decision support system, standardizing histopathological assessment.

## 1. Introduction

The microscopic evaluation of tissue by pathologists is the cornerstone of cancer diagnosis and an essential determinant of patient care pathways in oncology [1]. For ovarian carcinoma, a gynecological neoplasm with high worldwide mortality [2], this histopathological classification into one of five main subtypes, high-grade serous (HGSC), endometrioid (EC), clear cell (CCC), mucinous (MC), or low-grade serous (LGSC), is fundamental for guiding therapeutic strategies [3]. Each subtype exhibits distinct clinical behaviors and molecular profiles, making an accurate diagnosis essential for precision medicine [4].

However, this “gold standard” of diagnosis, which relies on human interpretation of morphology, faces a well-documented and significant challenge: interobserver variability. Studies have revealed that diagnostic agreement among pathologists for histological type can be as low as 60% [5], with reproducibility for even common subtypes like HGSC being only “fair” (Kappa coefficient of 0.34) in some cohorts [6]. This diagnostic inconsistency can directly impact patient stratification and therapeutic choices [7], creating an urgent clinical need for objective and reproducible tools that can support pathologists and standardize the diagnostic process [8].

In response to this need, the field of computational pathology has emerged, leveraging the digitization of whole-slide images (WSIs) to apply artificial intelligence (AI) as a powerful diagnostic aid [9]. These computational approaches promise to analyze tissue morphology with a level of consistency that can augment human assessment, improving reproducibility and uncovering quantitative patterns not apparent to the human eye [6]. Early successes have shown AI algorithms performing at a level comparable to human experts [10,11], paving the way for solutions to more complex challenges like automated cancer subtyping [12].

Despite the promise of AI, its application in this domain is hampered by a critical, unresolved challenge: the tendency of standard attention mechanisms to overfit via *attention concentration*, limiting model robustness. This study directly addresses this gap by developing and validating an AI tool that employs a direct regularization technique to provide a more generalizable and reproducible classification. The intended clinical role is as a “digital second reader” to augment and support the pathologist’s diagnosis, thereby enhancing consistency. Our objective is to evaluate the diagnostic accuracy of this tool using a public dataset of 538 whole-slide images. We employ a multiple instance learning (MIL) framework with an attention mechanism [13], which allows the model to mimic a pathologist’s workflow by identifying the most diagnostically relevant regions within a WSI. Crucially, this mechanism also generates attention maps, offering a layer of interpretability that allows the pathologist to verify the model’s reasoning—a crucial step toward building the trust required for clinical adoption [14]. It is also recognized that AI models can inadvertently perpetuate existing health inequalities if not carefully evaluated. While this study uses multi-center data to promote generalizability, we acknowledge the importance of future work to explicitly assess model fairness across different sociodemographic groups.

The main contributions of this work are threefold: (1) we develop and validate a high-performing deep learning model for ovarian cancer histotype classification that directly addresses the issue of interobserver variability; (2) we conduct a rigorous, systematic comparison of multiple attention-based MIL architectures, demonstrating that a stochastic attention mechanism provides a statistically significant performance advantage for this clinical application; and (3) we validate the model’s robustness and generalization on a challenging, independent molecular subtyping task. This paper is organized as follows: Section 2 provides a detailed review of the related literature to establish the context and research gap. Section 3 details the datasets, MIL models, and evaluation methods. Section 4 presents the classification performance results. Section 5 discusses the implications of our findings, study limitations, and directions for future research, followed by the conclusion in Section 6.

## 2. Related Work

### 2.1. The Clinical Imperative and the Challenge of Interobserver Variability in Ovarian Cancer Histotyping

The histopathological classification of ovarian carcinoma is a cornerstone of modern oncology, serving as a critical determinant of patient management and therapeutic strategy [2]. Ovarian cancer is not a monolithic entity but comprises at least five principal histotypes: high-grade serous (HGSC), low-grade serous (LGSC), endometrioid (EC), clear cell (CCC), and mucinous (MC) carcinoma. Each of these subtypes represents a distinct disease with unique molecular profiles, clinical behaviors, and prognoses, a realization that has shifted the field away from treating ovarian cancer as a single disease towards a more nuanced, histotype-specific management approach [15]. Consequently, an accurate and reproducible diagnosis is fundamental to the practice of precision medicine, directly informing decisions regarding surgical approaches, chemotherapy regimens, and the use of targeted therapies.

Despite its foundational role, the “gold standard” of microscopic evaluation by pathologists is encumbered by a well-documented and persistent challenge: significant interobserver variability [6]. This issue has been recognized for decades, with a seminal 1984 study by Hernandez et al. reporting a diagnostic agreement rate for histologic type of only 60% and for grade of 66% among pathologists reviewing the same cases [5]. The primary causes identified for these discrepancies were tumor cell heterogeneity and the use of differing subjective criteria for classification [5]. Even with the evolution of diagnostic criteria and the adjunctive use of immunohistochemistry (IHC), which has been shown to improve concordance, the problem remains significant [4]. More recent studies have reported reproducibility for even the most common subtype, HGSC, as being merely “fair,” with Cohen’s Kappa coefficients as low as 0.34 in some cohorts. This diagnostic inconsistency is not a minor academic concern; it directly translates to ambiguity in patient stratification and potential inconsistencies in treatment, creating an urgent clinical need for objective and reproducible tools that can support pathologists and standardize the diagnostic process [6]. The very foundation of personalized medicine in ovarian cancer—the ability to reliably stratify patients by histotype—is undermined by the subjectivity inherent in the primary diagnostic method. This implies that the potential benefits of histotype-specific therapies cannot be fully realized if the initial classification is inconsistent, justifying the pursuit of technologies that can introduce a higher degree of objectivity and reproducibility.

### 2.2. Computational Pathology and the Rise of Multiple Instance Learning (MIL)

In response to this clinical need, the field of computational pathology has emerged, leveraging the digitization of conventional glass slides into whole-slide images (WSI) to apply artificial intelligence (AI) as a powerful diagnostic aid [3,6]. These computational approaches promise to analyze tissue morphology with a level of consistency that can augment human assessment, improve reproducibility, and uncover complex quantitative patterns that are not apparent to the human eye [6]. The potential of AI to enhance diagnostic workflows has been demonstrated across numerous oncological domains, with algorithms achieving performance comparable to, and in some cases exceeding, that of human experts, thereby paving the way for solutions to more complex challenges such as automated cancer subtyping [3,12].

The unique characteristics of WSIs, however, present formidable technical challenges. Their gigapixel-scale resolution (e.g., images exceeding 60,000 × 40,000 pixels) makes direct processing by standard convolutional neural networks (CNNs) computationally infeasible due to GPU memory constraints. Furthermore, the process of obtaining exhaustive, fine-grained annotations (i.e., pixel-level or region-level labels) from expert pathologists is prohibitively labor-intensive and does not scale [13]. Consequently, the classification of WSIs is almost universally framed as a weakly supervised learning problem, for which multiple instance learning (MIL) has become the dominant paradigm [13]. In the MIL framework, each WSI is conceptualized as a “bag,” which is assigned a single slide-level label (e.g., “HGSC”). The bag contains thousands of unlabeled “instances,” which are smaller image patches tessellated from the WSI. The objective of the MIL model is to predict the bag-level label from this collection of unlabeled instances, thus circumventing the need for detailed spatial annotations.

This paradigm evolved significantly with the introduction of attention-based MIL (ABMIL) frameworks, most notably the Clustering-Constrained Attention Multiple Instance Learning (CLAM) model proposed by Lu et al. [13]. ABMIL employs an attention mechanism to learn the relative importance of each instance within the bag. This mechanism computes a slide-level representation as a weighted average of the instance features, where the weights are the learned attention scores [13]. This approach serves a critical dual purpose. First, it enables accurate slide-level classification by intelligently aggregating the most informative features from across the WSI. Second, it produces an attention map, or heatmap, which visually highlights the subregions that the model deemed most important for its prediction. This offers a crucial layer of interpretability, allowing a pathologist to verify that the model’s reasoning is based on diagnostically relevant morphology, a key step toward building the trust required for clinical adoption [14].

### 2.3. A Critical Analysis of ABMIL: Limitations and the Emerging Research Frontier

Despite their success and widespread adoption, a growing body of literature has begun to critically examine the inherent limitations of standard attention mechanisms in the context of computational pathology. A primary issue is that of attention concentration, where the model learns to focus excessively on a very small subset of the most discriminative instances while effectively ignoring the rest of the tissue information present in the WSI. This behavior is strongly correlated with model overfitting. The model identifies a simple, low-effort strategy to minimize its training loss by relying on a few “easy” or highly salient morphological patterns—for example, a region of overtly high-grade tumor. This behavior is a form of pathological “shortcut learning”; the model learns to find the most obvious signal rather than developing a holistic and robust understanding of the disease’s morphological presentation. A human pathologist’s diagnosis, in contrast, integrates information from the overall tissue architecture, the interface between tumor and stroma, and the heterogeneity across the entire lesion. By taking a shortcut, the model fails to learn from the full spectrum of features, including more subtle or “hard-to-classify” instances, which ultimately limits its robustness and ability to generalize to unseen data, particularly cases with atypical presentations or from different medical centers.

Further deepening this critique, recent work has questioned the fundamental assumption that attention scores provide a faithful explanation of a model’s decision-making process [16]. A high attention score assigned to a patch does not necessarily guarantee a high contribution to the final prediction, as the slide-level output is a complex, non-linear function of both the attention-weighted feature aggregation and the subsequent classification head. Indeed, interventional studies have demonstrated that regions of high attention can sometimes have a neutral or even counterintuitive (negative) impact on the model’s output, and that attention maps can be manipulated without altering the final prediction. This misalignment between what the model appears to be “looking at” and what it is actually using for its decision undermines the trustworthiness of attention-based explanations. This gap between interpretability and faithfulness represents a significant hurdle for the clinical translation of these models, where understanding the “why” behind a prediction is as important as the prediction itself [14].

### 2.4. The Research Gap: The Need for Direct Regularization of the Attention Mechanism

The existing literature thus presents a clear narrative: there is a compelling clinical need for objective and reproducible ovarian cancer histotyping that standard pathology cannot fully meet [5]. Computational pathology, particularly through the use of ABMIL, offers a powerful solution [13]. However, this state-of-the-art approach is hampered by a critical, unresolved challenge: the tendency of the attention mechanism to concentrate and overfit, a behavior that limits model robustness and calls into question the faithfulness of its explanations. In response, various architectural modifications have been proposed. For instance, some works have explored multi-branch attention (MAB) to allow different attention heads to specialize in features for different classes, a strategy hypothesized to be beneficial in multiclass settings. Other approaches have focused on refining instance representations through contrastive or prototype learning. However, these methods primarily address the problem by adding architectural complexity around the attention mechanism rather than modifying the behavior of the mechanism itself.

This reveals a distinct research gap: there is a need for methods that *directly regularize the attention mechanism* to explicitly combat the phenomenon of attention concentration. The field requires a technique that forces the model to learn from a wider and more diverse set of discriminative features across the WSI, rather than permitting it to over-rely on a few highly salient instances. Such a regularization strategy would, in theory, promote the learning of a more robust and generalizable representation of tissue morphology. This work aims to fill this gap by proposing and systematically evaluating a stochastic attention mechanism as a direct and efficient form of regularization designed to mitigate attention concentration, thereby improving both the accuracy and generalization of MIL models for the clinically vital task of ovarian cancer histotype classification.

## 3. Materials and Methods

This study is a retrospective diagnostic accuracy study using publicly available data. All data were collected prior to the initiation of our analysis.

### 3.1. Participants and Data Sources

#### 3.1.1. Histological Subtyping Dataset: UBC-OCEAN

**Figure 1:**
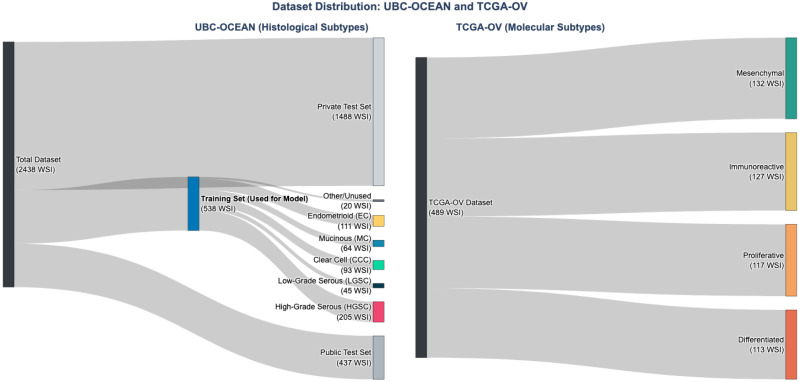
Sankey diagrams illustrating the flow and composition of participants in the two datasets used in this study. (**Left**) The UBC-OCEAN dataset, showing the flow from the total images to the training set used for model development, which is further broken down by histological subtype. (**Right**) The TCGA-OV dataset, showing the distribution of the 489 high-grade serous carcinoma cases across the four consensus molecular subtypes used for the generalization task.

For the primary task of histological classification, we utilized the public training data from the University of British Columbia Ovarian Cancer (UBC-OCEAN) challenge [17]. This dataset represents a crucial benchmark for the field as it is the largest and most diverse public collection of ovarian cancer histopathology images, sourced from 24 different clinical centers, primarily through the Ovarian Tumor Tissue Analysis (OTTA) consortium [4]. Its multi-center nature introduces significant real-world variability in patient demographics, tissue processing, and staining, providing a rigorous test for the generalizability of any AI model. The full challenge dataset comprises 2438 images, but our study was conducted exclusively on the officially provided training set of 538 images. This set contains the five main histotypes and was used in its entirety for our experiments. The data distribution is visualized in Figure 1. The participants constitute a convenience series, comprising all available cases within the public training dataset.

#### 3.1.2. Molecular Subtyping Dataset: TCGA-OV

To evaluate the model’s ability to generalize, we selected a second, independent public dataset: high-grade serous carcinoma cases from The Cancer Genome Atlas Ovarian Cancer (TCGA-OV) cohort [15]. This task tests the model’s capacity to identify subtle morphological features that correlate with underlying molecular states. Specifically, we aimed to classify the 489 WSIs into the four consensus molecular subtypes previously established by transcriptomic analysis [15]. The distribution of these subtypes is shown in Figure 1. The original patient data for both the OTTA and TCGA cohorts were collected over several years prior to their respective publications. For this secondary analysis, the computational work was conducted between 20 October 2024, and 15 March 2025.

#### 3.1.3. Inclusion and Exclusion Criteria

For the UBC-OCEAN dataset, all 538 whole-slide images from the official training set were initially included. The inclusion criterion was a definitive diagnosis of one of the five major ovarian carcinoma histotypes (HGSC, EC, CCC, MC, LGSC). For the TCGA-OV dataset, inclusion was limited to WSIs from cases with a primary diagnosis of high-grade serous ovarian carcinoma and for which a consensus molecular subtype label was available. We performed a quality control check using the HistoQC library [18]. For the UBC-OCEAN dataset, all 538 slides passed this quality control. For the TCGA-OV dataset, we excluded 12 slides due to significant artifacts such as pen markings, which resulted in a final analysis set of 477 slides. No information on treatments received by the patients was available in these public datasets, and patient demographic data was not provided, precluding an analysis of model fairness across different subgroups.

#### 3.1.4. Data Preprocessing and Patch-Level Statistics

To prepare the WSIs for the MIL framework, we implemented a standardized preprocessing pipeline. This pipeline first identifies the primary tissue regions on each slide using an automated segmentation algorithm (see Section 3.2.1 for details), effectively filtering out background and slide artifacts. These segmented tissue areas are then tessellated into non-overlapping 256 × 256 pixel patches at 20× magnification. This process transforms each high-resolution WSI (a “bag”) into a collection of thousands of smaller image patches (“instances”), which are the fundamental units of analysis for the MIL models. Following tessellation, we performed a final quality control step to discard patches with excessive blur or staining artifacts. Table 1 provides detailed descriptive statistics for both the UBC-OCEAN and TCGA-OV datasets after this preprocessing. It summarizes the number of slides (bags) for each subtype and presents the mean, standard deviation, and estimated total number of patches (instances) generated, illustrating the scale of the data at the instance level.

### 3.2. Test Methods

#### 3.2.1. Index Test: AI-Based MIL Model

The index test is the AI-based classification tool developed in this study. Given that WSIs are massive images, direct processing is computationally infeasible. These images are often in the gigapixel scale; for instance, a slide scanned at 20× magnification can easily exceed 60,000 × 40,000 pixels. We adopted a patch-based approach. Each WSI first underwent tissue segmentation to identify diagnostically relevant regions. We performed tissue segmentation using a multi-step algorithm we adapted from the pipeline proposed by Lu et al. [13]. First, we convert the WSI from the RGB to the HSV color space to better isolate color and intensity information. We then apply a median filter to the saturation channel to suppress noise while preserving edges. Subsequently, we generate a binary mask by applying a threshold to this filtered channel and identify the primary tissue regions by detecting contours in this binary mask. We apply a two-stage filtering process: we filter initial contours based on a minimum area threshold to eliminate small, non-tissue artifacts, and similarly filter holes within the valid tissue contours to ensure we only exclude substantive empty regions. We detail the complete algorithm in Algorithm 1. For full transparency and reproducibility, we have made the source code for this implementation available in the study’s public repository. These regions were then tessellated into non-overlapping 256 × 256 pixel patches at 20 × magnification. For each patch, we extracted a 512-dimensional feature vector using CONCH [19], a foundation model pre-trained on pathology data. These feature vectors served as the input for our MIL models, which are detailed in Section 3.3.

The final output of the model is a set of probabilities, one for each class. A slide was assigned to the class with the highest probability; this maximum probability rule served as the pre-specified threshold for classification.
**Algorithm 1** Tissue segmentation from WSI  1:**procedure** SegmentTissue($WSI,seg\_level,s_{thresh},m_{thresh},a_{thresh}$)  2:    $I_{RGB}\leftarrow \mathrm{ReadRegion}\left(WSI,\mathrm{level}=seg\_level\right)$  3:    $I_{HSV}\leftarrow \mathrm{ConvertToHSV}\left(I_{RGB}\right)$  4:    $S_{channel}\leftarrow I_{HSV}\left[:,:,1\right]$            ▷ Extract saturation channel  5:    $S_{filtered}\leftarrow \mathrm{MedianFilter}\left(S_{channel},\mathrm{kernel}\_\mathrm{size}=m_{thresh}\right)$  6:    $I_{binary}\leftarrow \mathrm{BinaryThreshold}\left(S_{filtered},\mathrm{threshold}=s_{thresh}\right)$  7:    $contours,hierarchy\leftarrow \mathrm{FindContours}(I_{binary})$  8:    $filtered\_contours,filtered\_holes\leftarrow \mathrm{FilterContoursByArea}(contours,hierarchy,a_{thresh})$  9:    scaled_contours←ScaleContoursToLevel0(filtered_contours,seg_level)10:    scaled_holes←ScaleContoursToLevel0(filtered_holes,seg_level)11:    **return** scaled_contours,scaled_holes12:**end procedure**

#### 3.2.2. Reference Standard

The reference standard for this study was the slide-level diagnostic label provided within each public dataset. For the UBC-OCEAN dataset, this was the histological subtype (e.g., HGSC, EC), and for the TCGA-OV dataset, it was the molecular subtype (e.g., immunoreactive). These labels were established by the expert pathologists and researchers of the OTTA and TCGA consortia, respectively, and are considered the best available method for establishing the ground truth for this computational task.

#### 3.2.3. Blinding

The AI model (the index test) was blinded to the reference standard labels during the evaluation phase of each cross-validation fold. The predictions were generated based solely on the image features. The reference standard labels were established by the source consortia years before this study and without any knowledge of our index test.

### 3.3. Machine Learning Model: Multiple Instance Learning

**Figure 2:**
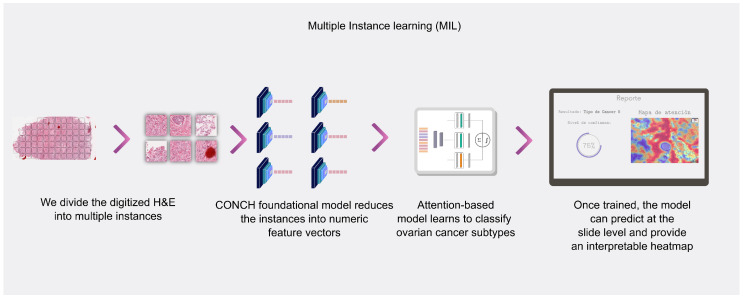
Workflow of the multiple instance learning (MIL) diagnostic tool (the index test). The WSI is divided into instances, features are extracted for each, and an attention-based model predicts the subtype while generating an attention map for interpretability.

The MIL paradigm is fundamental to this work. The task of the MIL model is to infer the slide-level diagnosis (the “bag” label) from the set of its constituent patches (the “instances”), as illustrated in Figure 2. For a detailed algorithmic description of each model, please refer to Appendix A.

We implemented and evaluated models based on the CLAM architecture [13]. This architecture is distinguished by its use of a gated attention mechanism, which weights the importance of each instance for the bag-level prediction. This allows the model to learn to focus on the most informative patches, mimicking a pathologist’s diagnostic process. Furthermore, CLAM incorporates an instance-level clustering loss, which encourages a clear separation between candidate instances of different classes, thereby improving both performance and interpretability. All models first transform the input features through a fully connected layer with a ReLU activation function. The resulting transformed features are then fed into the specific attention or pruning module.

#### 3.3.1. Single Attention Branch (SAB)

This is the foundational CLAM model. Its core is a gated attention network with a single branch. For a bag of transformed instance features $H=\{h_{1},\ldots ,h_{N}\}$, where $h_{i}\in R^{L}$, the network computes an attention score $a_{i}$ for each instance. This is achieved through two parallel linear transformations of $h_{i}$: one followed by a tanh activation and the other by a σ (sigmoid) activation. The sigmoid acts as a gate, modulating the information from the tanh branch. The element-wise product of these two outputs is then passed through a final linear layer to produce the scalar attention score. This mechanism allows for a more complex and nuanced weighting of instances compared to a simpler attention network. The final slide-level representation, *M*, is a weighted average of the instance features using these attention scores, which is then used for classification as per Equation (1):

(1) $$\begin{array}{cc} a_{i} & =w^{T}\left(\tanh\left(Vh_{i}^{T}\right)⊙\sigma \left(Uh_{i}^{T}\right)\right)\\ \alpha_{i} & =\frac{\exp(a_{i})}{\sum_{j=1}^{N}\exp\left(a_{j}\right)}\\ M & =\sum_{i=1}^{N}\alpha_{i}h_{i}\\ \hat{y} & =\mathrm{Classifier}(M) \end{array}$$

where w,V, and *U* are learnable parameters of the linear layers, and ⊙ denotes element-wise multiplication.

#### 3.3.2. Multiple Attention Branches (MAB)

In contrast to the SAB model, which uses a single, general attention network to identify all regions of interest, the MAB model employs *C* parallel attention branches, where *C* is the number of classes. Theoretically, this allows each branch to specialize in identifying morphological patterns specific to one class. This specialization is hypothesized to improve performance in multiclass settings by disentangling class-specific features, preventing a single attention mechanism from having to model all subtypes simultaneously. Architecturally, this means the final layer of the attention network outputs *C* scores for each instance instead of one. This results in *C* distinct attention-weighted slide representations, $M_{c}$, one for each class. Each $M_{c}$ is then fed into its own dedicated linear classifier. The final prediction is based on the combined outputs, as shown in Equation (2):

(2) $$\begin{array}{cc} a_{i,c} & =w_{c}^{T}\left(\tanh\left(V_{c}h_{i}^{T}\right)⊙\sigma \left(U_{c}h_{i}^{T}\right)\right)\\ M_{c} & =\sum_{i=1}^{N}\frac{\exp(a_{i,c})}{\sum_{j=1}^{N}\exp\left(a_{j,c}\right)}h_{i}\\ \hat{y} & =\mathrm{softmax}(\left[{\mathrm{Classifier}}_{1}\left(M_{1}\right),\ldots ,{\mathrm{Classifier}}_{C}\left(M_{C}\right)\right]) \end{array}$$

#### 3.3.3. Dynamic Instance Pruning (DIP)

This approach introduces a preliminary filtering step before the main attention mechanism. It uses a lightweight scoring network—a simple two-layer perceptron (Linear -> ReLU -> Linear) to assign a relevance score to each raw instance feature. Only the top-*K* instances with the highest scores are retained. This pruned set is then processed by the standard SAB model. The rationale is twofold: first, it improves computational efficiency by reducing the number of instances the more complex attention network must process. Second, it acts as a noise reduction mechanism by discarding potentially irrelevant or uninformative instances early, allowing the attention model to focus its capacity on a smaller, more promising subset of the data, as defined in Equation (3).

(3) $$s_{i}=f_{\mathrm{scorer}}\left(h_{i}\right),\;H_{\mathrm{pruned}}=\mathrm{TopK}\left(\left\{\left(h_{1},s_{1}\right),\ldots ,\left(h_{N},s_{N}\right)\right\}\right)$$

#### 3.3.4. Stochastic Attention (SA)

This variant introduces a regularization technique into the SAB model to improve generalization. During training, after computing the attention probabilities $\alpha_{i}$ for all instances, the model does not use all of them for the weighted average. Instead, it stochastically samples a small subset of *k* instances, where the probability of selecting each instance is proportional to its attention weight $\alpha_{i}$. The slide representation $M_{\mathrm{train}}$ is then a re-normalized, weighted average of this small, sampled subset. This process acts as a regularizer, akin to dropout, by preventing the model from over-relying on a few specific high-attention instances in each bag. It forces the model to learn from a wider variety of discriminative features, thus improving its robustness. During evaluation, the model reverts to the deterministic SAB method, using all instances for the aggregation. We investigated this with *k* values of 4, 16, and 32 (referred to as SA-4, SA-16, and SA-32, respectively).

### 3.4. Analysis

#### 3.4.1. Sample Size

This study utilized all available cases from the public UBC-OCEAN (n = 538) and TCGA-OV (n = 489, prior to quality control) cohorts. The sample size was therefore determined by the content of these established repositories.

#### 3.4.2. Handling of Missing and Indeterminate Data

The analysis was performed on a complete-case basis. An automated preprocessing and quality control pipeline was established to handle WSIs; any slide that failed this pipeline (e.g., due to file corruption or significant artifacts) would be excluded. While all slides from the UBC-OCEAN dataset were processed successfully, 12 slides from the TCGA-OV dataset were excluded due to quality control issues, as detailed in Section 3.1.3.

#### 3.4.3. Experimental Setup and Training

We evaluated the models on two distinct classification tasks: histological subtyping and molecular subtyping. We employed a repeated random sub-sampling validation strategy. The process was repeated 10 times. In each of the 10 runs, the dataset was randomly partitioned into a training set (75%), a validation set (12.5%), and a test set (12.5%). Final performance metrics were averaged across the 10 runs. A similar scheme was applied to the TCGA-OV dataset. To address class imbalance in both tasks, we used weighted sampling during training. Models were trained for a maximum of 50 epochs using the Adam optimizer with a learning rate of 2 × 10^−4^. We applied a bag-level cross-entropy loss function and standard regularization techniques, including dropout. Early stopping was implemented with a patience of 10 epochs, monitoring the validation loss to prevent overfitting. We conducted the experiments on the Google Cloud Platform using NVIDIA A100 GPUs with 40 GB of memory. We used a batch size of 1 because each WSI (bag) contains thousands of patches (instances), and all instance features for a single bag must be loaded into memory for the attention mechanism, making the process memory-intensive. The source code is available on GitHub: https://github.com/dagoPA/cancer-de-ovario (accessed on 30 August 2025).

#### 3.4.4. Statistical Performance Evaluation

We quantified diagnostic performance using metrics appropriate for multiclass classification on imbalanced datasets.

Cohen’s Kappa (κ) was chosen to measure inter-rater agreement corrected for chance. This metric is particularly relevant as it directly contrasts with the low agreement rates often seen in manual pathological review.

Balanced Accuracy was selected for its robustness as a performance measure on imbalanced datasets, as it avoids inflation from the majority class by averaging the recall of each class.

Receiver Operating Characteristic (ROC) Curve and Area Under the Curve (AUC-ROC) were used to illustrate diagnostic ability. We report the macro-average (unweighted mean of the AUC for each class).

Precision–Recall (PR) Curve and Area Under the Curve (AUC-PR) were included as they are particularly informative for imbalanced datasets. We report the macro-averaged AUC-PR.

Confidence Intervals and Statistical Stability. We calculated 95% confidence intervals (95% CIs) using non-parametric bootstrap resampling (1000 repetitions) to estimate the uncertainty and stability of our metrics.

#### 3.4.5. Statistical Comparison of Models

To rigorously compare the performance of the different MIL models, we employed non-parametric statistical tests, which do not assume a normal distribution of the data and are therefore well-suited for comparing model performance metrics across cross-validation folds [20].

Wilcoxon Signed-Rank Test for Pairwise Comparisons. For direct comparisons between two models, we used the Wilcoxon signed-rank test. This test is appropriate for this study because the performance metrics (e.g., balanced accuracy) for each model are generated from the same cross-validation folds, resulting in paired samples. As a non-parametric method, it does not assume that the differences in performance between models follow a normal distribution, making it a robust choice. The test evaluates the null hypothesis that the median difference between the paired performance scores is zero. A low p-value indicates a statistically significant difference, suggesting that one model consistently outperforms the other across the data folds.

Friedman and Nemenyi Tests for Overall Comparison. To compare all models simultaneously across the multiple cross-validation folds, we first applied the Friedman test. This is a non-parametric equivalent of the repeated measures ANOVA and tests the null hypothesis that there are no differences between the models’ performances. Since the Friedman test indicated a significant overall difference in our experiments, we proceeded with the Nemenyi post-hoc test for all pairwise comparisons. The Nemenyi test calculates a single critical difference (CD) value. The performance of two models is considered significantly different if their average rank across the folds differs by at least the CD. The results of this analysis are visualized using a critical difference (CD) diagram, which provides a clear visual summary of which models are statistically distinguishable from one another.

## 4. Results

To develop a tool that could reliably address diagnostic variability, we systematically evaluated our models, first on the primary task of histological classification on 538 WSIs. As detailed in Table 2, the stochastic attention model with k=4 (SA-4) demonstrated the highest overall performance. A key finding was that all attention-based models consistently yielded higher performance metrics than the non-attention-based dynamic instance pruning (DIP) model, underscoring the importance of the attention mechanism for this diagnostic task. In direct contrast with the low inter-rater agreement (Kappa coefficients as low as 0.34) reported in manual assessment [6], our best model achieved a Cohen’s Kappa of 0.791, alongside a balanced accuracy of 0.854. This highlights the model’s potential to provide a highly reproducible classification standard.

To further assess the robustness and generalization capabilities of our proposed models, we evaluated their performance on the more challenging task of classifying molecular subtypes from morphology alone within the TCGA-OV dataset of 489 WSIs. As expected, absolute performance was lower than on the primary task, but the relative performance trend held: the SA-4 model again achieved the highest performance metrics (Table 3). This consistently higher performance on a distinct and more difficult problem confirms that the architecture generalizes effectively and that the stochastic attention mechanism provides a robust advantage.

To quantify the statistical significance of these performance differences, we performed the statistical comparison tests detailed in the Methods section. The Wilcoxon signed-rank test was used for pairwise comparisons, confirming that the SA-4 model achieves a statistically significant improvement in balanced accuracy compared to all other models in both the histological and molecular subtyping tasks (Table 4).

**Figure 3:**
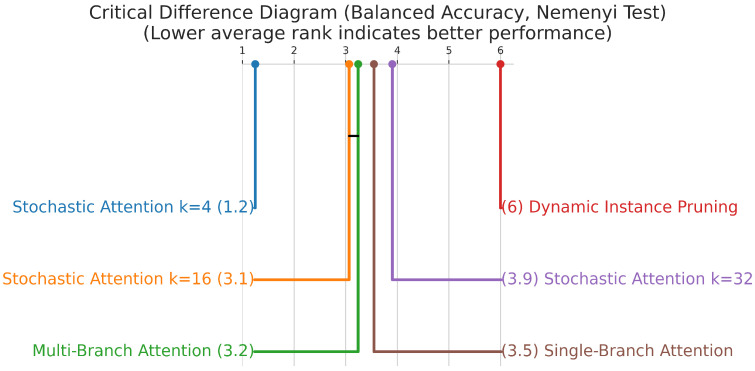
Critical difference diagram for model comparison on the histological subtyping task. The diagram plots the average rank of each model (lower is better). The stochastic attention k=4 model is positioned with the best rank and its performance is statistically significant compared to the other models (Nemenyi test, α=0.05).

**Figure 4:**
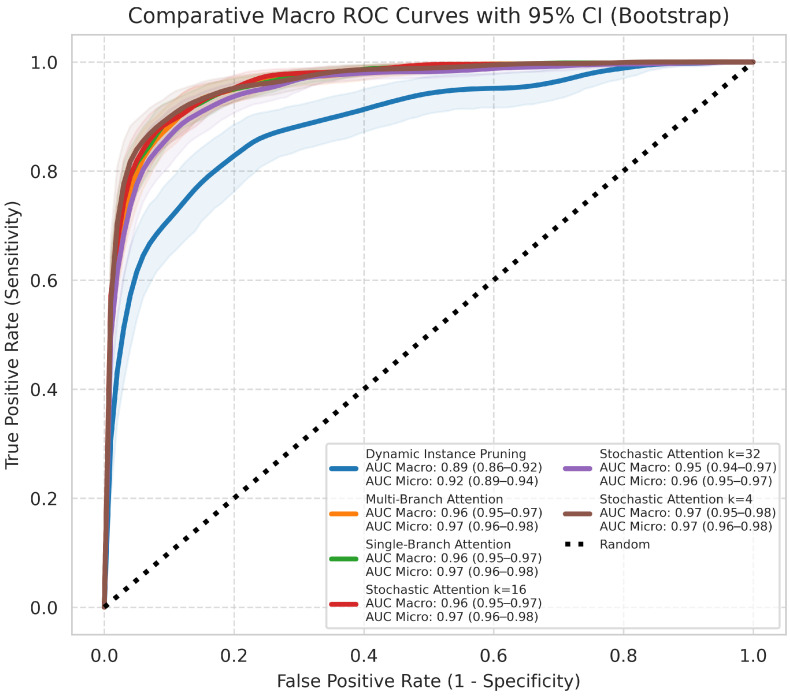
Comparative macro-average ROC curves with 95% CI for the histological subtyping task. The curves for the attention-based models are clustered in the top-left, indicating higher performance. The legend reports the macro-averaged AUC-ROC for each model.

**Figure 5:**
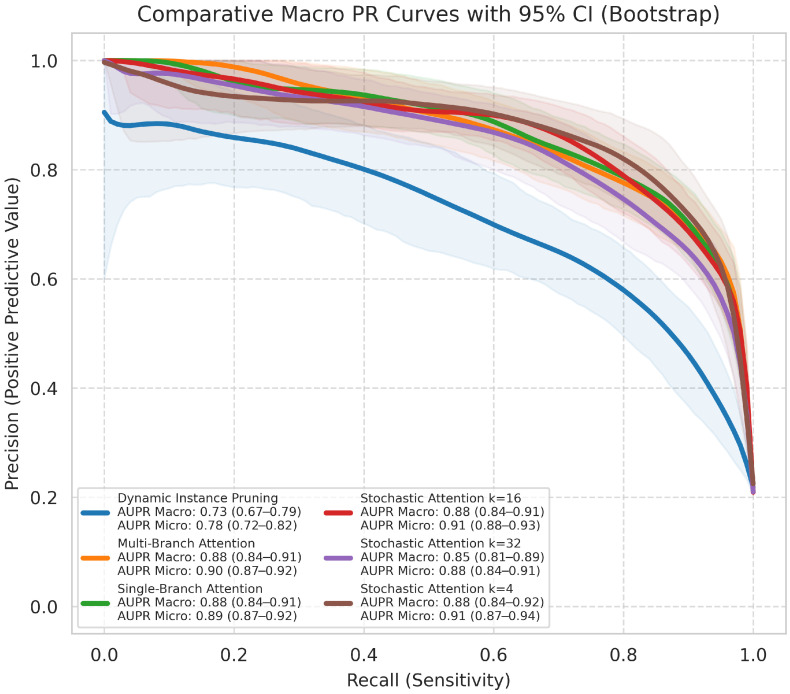
Comparative macro-average precision–recall (PR) curves with 95% CI for the histological subtyping task. This plot is informative for imbalanced datasets. Curves closer to the top-right indicate better performance.

**Figure 6:**
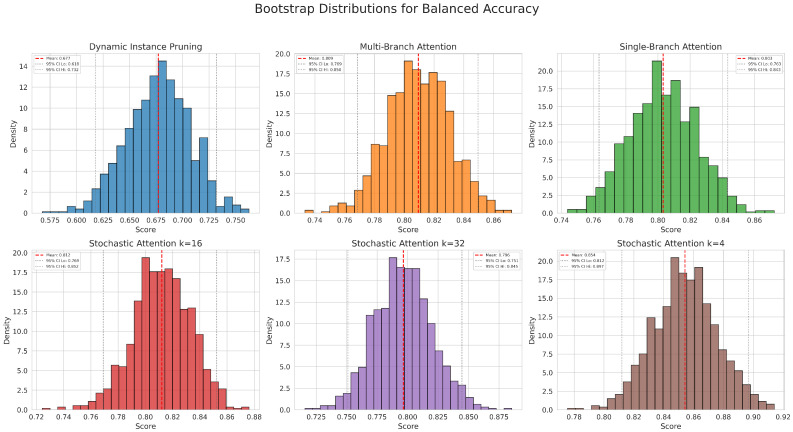
Bootstrap histograms of balanced accuracy for the histological subtyping task. These plots show the stable distribution of the balanced accuracy metric for each model, confirming the robustness of the performance estimates.

**Figure 7:**
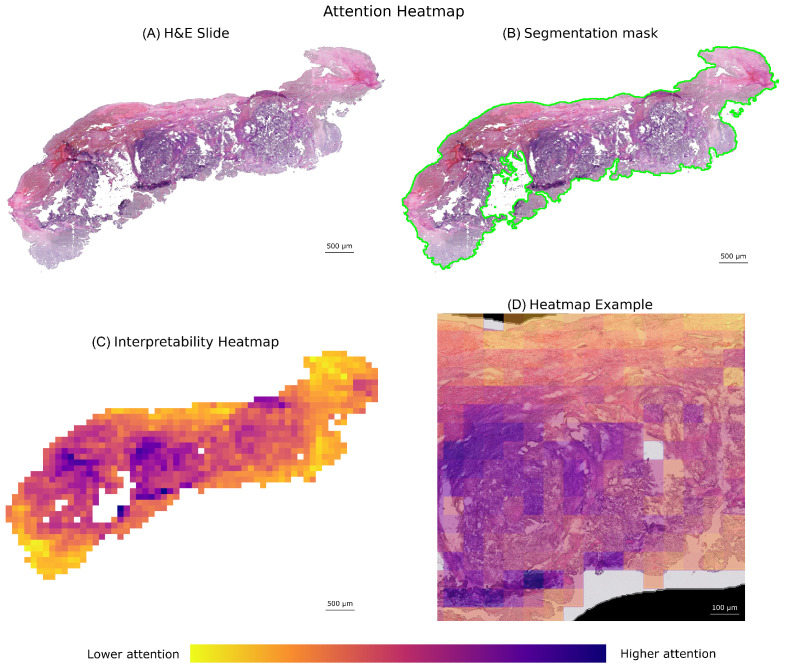
Visual explanation of the attention mechanism for model interpretability. This figure illustrates the process from the raw whole-slide image to the generated attention heatmap, providing insights into the model’s decision-making. (**A**) H&E Slide: The original histopathology whole-slide image (WSI) before any processing, with a scale bar included for reference. (**B**) Tissue Segmentation Mask: The segmented tissue region of the WSI, highlighted with a green outline, indicating the diagnostically relevant area from which patches were extracted for analysis. This step ensures the model focuses only on tissue. (**C**) Interpretability Heatmap: A visual representation of the model’s attention scores across the segmented tissue. The included color bar indicates the range of attention scores, where warmer colors (e.g., purple) indicate regions of higher importance or “attention” given by the model for its classification decision, while cooler colors (e.g., yellow) indicate less important regions. (**D**) Heatmap Overlay Example: A magnified view showing the interpretability heatmap (from panel (**C**)) overlaid onto the corresponding region of the H&E slide (from panel (**A**)). This overlay provides a crucial visual explanation for pathologist review, highlighting the specific morphological features or tissue patterns the model focused on for its prediction, thereby fostering trust and aiding in diagnostic verification.

The critical difference diagram (Figure 3), which visualizes the results of the Nemenyi post-hoc test, further confirms the superior performance of the SA-4 model for the primary histological subtyping task. Its average rank is significantly better than all other models, as it is not connected to any other model group within the critical difference threshold. The macro-average ROC and PR curves (Figure 4 and Figure 5) further illustrate the high discriminative capacity of the attention-based models. The bootstrap analysis (Figure 6) demonstrates the stability of the balanced accuracy metric, and the generated attention heatmaps (Figure 7) provide a crucial layer of interpretability for pathologists.

### Computational Complexity

In terms of computational complexity, our final SA-4 model has approximately 1.5 million trainable parameters. The full pipeline from WSI to prediction, including preprocessing and inference, took an average of 12 min per slide on an NVIDIA A100 GPU, with model training averaging 40 min per run.

In binary (one-vs.-rest) tasks for the primary dataset, the stochastic attention k=4 model also demonstrated strong discrimination, achieving balanced accuracies from 0.891 for EC to an exceptional 0.961 for CCC, with macro-average AUC-ROC values greater than 0.965 for all subtypes. This indicates that the features learned are effective for both fine-grained multiclass distinction and robust individual subtype identification.

## 5. Discussion

This study presents a high-performing AI tool that directly addresses a key unmet need in diagnostic pathology: the significant interobserver variability in the classification of ovarian carcinoma histotypes. Where traditional microscopic assessment can yield diagnostic agreement as low as 60% [5], our tool provides a highly consistent and accurate classification, achieving a balanced accuracy of 0.854 and a strong Kappa score of 0.791. The robustness of this approach was further validated by its high performance on the distinct and more difficult task of molecular subtype classification from histology, confirming its generalization capabilities across problems of varying complexity.

The performance of our model is comparable to other state-of-the-art methods evaluated on the UBC-OCEAN dataset. For instance, the top-performing solutions in the OCEAN challenge, which utilized this dataset, achieved balanced accuracies between 58% and 66% on the private test set [17]. The winning team, Phikon, reported a mean AUC of 0.855 across multiple biomarker prediction tasks with their initial model [21]. While a direct comparison is challenging due to different evaluation metrics and test sets, it is noteworthy that many top-performing approaches relied on complex model ensembling or computationally intensive feature extractors. In contrast, our SA-4 model’s balanced accuracy of 0.854 on the public training set demonstrates highly competitive performance achieved by directly addressing the issue of attention concentration within a standard framework. This highlights the effectiveness of stochastic attention as a targeted and efficient regularization strategy, rather than relying on increased architectural complexity.

The success of our approach lies in a synergistic combination of methods. The MIL framework is ideally suited to the weakly supervised nature of WSI analysis. The attention mechanism, acting as a “digital magnifying glass,” intelligently identifies diagnostically relevant regions, and the higher performance of the stochastic variant (k=4) across both tasks highlights its power as a regularizer that improves generalization. Finally, leveraging features from the pathology-specific CONCH foundation model provided a high-quality representational starting point for our classifier.

A key aspect for clinical translation is the integration of such a tool into the pathology workflow. We envision this AI model not as a replacement for the pathologist, but as a “digital second reader” or triage tool. The attention heatmaps (Figure 7) provide an additional layer of explainability by visualizing the regions the model deems important for its prediction. This allows for pathologist verification, though we acknowledge that this shows *where* the model is looking, not necessarily *why* in pathologically semantic terms. This visual feedback is integral to a human-in-the-loop system, building trust and augmenting, rather than replacing, human expertise. This could increase diagnostic throughput and, most importantly, improve diagnostic consistency across institutions.

However, several limitations must be addressed before clinical adoption. A primary limitation of this study is its retrospective nature. Although we used large, multi-center public datasets, prospective validation on new, real-world clinical data is an essential next step to confirm the model’s generalizability and clinical utility before any potential adoption. A significant limitation is the lack of patient demographic data and detailed information about the contributing centers, which prevented an analysis of model fairness or performance disparities. Future studies must prioritize the use of datasets that allow for such analysis to ensure that AI tools are equitable. Second, our study was limited to the five main ovarian cancer subtypes and did not include rare histotypes or borderline tumors, which represent important future work. Third, while attention maps provide a degree of interpretability by showing *where* the model is looking, they do not fully explain *why* in pathologically meaningful terms [22]. This “black box” nature remains a hurdle for clinical trust that the field must overcome [14]. Finally, in a clinical setting, a protocol would be needed to handle slides that fail the quality control pipeline, for instance, by flagging them for manual review.

Future work must be directed at surmounting these barriers. Prospective studies in real-world clinical settings are needed to quantify the tool’s impact on diagnostic accuracy, efficiency, and pathologist confidence. Integrating our morphological model with other data modalities, such as genomics or proteomics, could lead to more powerful multi-modal diagnostic systems [23,24]. Finally, advancing explainable AI (XAI) beyond simple heatmaps is essential. Developing methods that can articulate the model’s reasoning in terms verifiable by pathologists will be crucial for building trust and facilitating seamless clinical adoption [25]. Critically, future studies must prioritize the collection of demographic data to allow for rigorous evaluation of model fairness and to ensure that these powerful tools reduce, rather than exacerbate, health disparities. Future work could also explore the integration of Transformer-based architectures, which have shown promise in other computational pathology tasks [26,27] and may offer alternative approaches to feature representation.

## 6. Conclusions

In this study, we developed and rigorously validated an AI-based diagnostic support tool that effectively classifies ovarian carcinoma histotypes with a level of accuracy and consistency that directly addresses the documented variability of manual microscopic assessment. Our best-performing model demonstrated robust generalization on a secondary, more challenging molecular subtyping task, a result attributed to a combination of attention-based learning and features from a pathology-specific foundation model. These findings highlight the potential of AI to serve as a powerful complementary tool in computational pathology, bringing greater objectivity to histopathological evaluation and augmenting the pathologist’s workflow. For reliable clinical application, this tool requires further external validation and continued development in explainable AI to foster clinical trust and ensure its seamless integration into the diagnostic process.

## Figures and Tables

**Table 1:** Descriptive statistics of the processed WSI datasets. The table shows the number of slides (bags) per subtype, along with the mean and total estimated number of 256 × 256 pixel patches (instances) generated after preprocessing.

Dataset	Subtype	Slides (Bags)	Mean Patches per Slide	Total Patches
**UBC-OCEAN**	High-Grade Serous (HGSC)	205	3550	731,300
	Endometrioid (EC)	111	3610	429,590
	Clear Cell (CCC)	93	3850	323,400
	Mucinous (MC)	64	4120	387,280
	Low-Grade Serous (LGSC)	45	3240	113,400
TCGA-OV *	Mesenchymal	132	3730	529,660
	Immunoreactive	127	3480	441,960
	Proliferative	117	3880	453,960
	Differentiated	113	3520	362,560

* For the TCGA-OV dataset, numbers correspond to the original cohort of 489 slides. The final analysis was performed on 477 slides after quality control.

**Table 2:** Diagnostic performance of MIL models in the multiclass classification of ovarian carcinoma histological subtypes (UBC-OCEAN dataset).

Model	Cohen’s Kappa	Balanced Accuracy	AUC-ROC (Macro)	AUC-ROC (Micro)	AUC-PR (Macro)	AUC-PR (Micro)
DIP	0.587 (0.525–0.651)	0.676 (0.619–0.731)	0.894 (0.864–0.921)	0.916 (0.896–0.935)	0.720 (0.668–0.789)	0.776 (0.724–0.825)
MAB	0.742 (0.690–0.789)	0.808 (0.763–0.849)	0.961 (0.950–0.971)	0.967 (0.958–0.976)	0.878 (0.843–0.909)	0.896 (0.867–0.922)
SAB	0.737 (0.690–0.780)	0.803 (0.759–0.844)	0.962 (0.952–0.972)	0.966 (0.957–0.976)	0.877 (0.841–0.909)	0.893 (0.864–0.919)
SA-16	0.758 (0.711–0.803)	0.811 (0.770–0.850)	0.964 (0.953–0.973)	0.970 (0.960–0.979)	0.873 (0.840–0.908)	0.907 (0.881–0.930)
SA-32	0.731 (0.684–0.778)	0.796 (0.749–0.842)	0.953 (0.939–0.965)	0.958 (0.946–0.969)	0.850 (0.809–0.888)	0.876 (0.839–0.906)
**SA-4**	**0.791 (0.739–0.835)**	**0.854 (0.809–0.898)**	**0.965 (0.952–0.976)**	**0.970 (0.959–0.981)**	**0.883 (0.839–0.924)**	**0.906 (0.873-0.937)**

Bold indicates the best-performing model.

**Table 3:** Generalization performance on the secondary task of molecular subtype classification (TCGA-OV). The table details the performance of the models on the more challenging task of predicting molecular subtypes from histology, using the independent TCGA-OV dataset to evaluate model robustness and generalization capability.

Model	Cohen’s Kappa	Balanced Accuracy	AUC-ROC (Macro)	AUC-ROC (Micro)	AUC-PR (Macro)	AUC-PR (Micro)
DIP	0.485 (0.430–0.540)	0.602 (0.548–0.656)	0.745 (0.700–0.790)	0.798 (0.759–0.837)	0.553 (0.501–0.605)	0.615 (0.568–0.662)
MAB	0.565 (0.510–0.620)	0.668 (0.614–0.722)	0.805 (0.760–0.850)	0.841 (0.805–0.877)	0.648 (0.596–0.700)	0.695 (0.649–0.741)
SAB	0.558 (0.503–0.613)	0.661 (0.607–0.715)	0.798 (0.753–0.843)	0.835 (0.799–0.871)	0.641 (0.589–0.693)	0.688 (0.642–0.734)
SA-16	0.582 (0.527–0.637)	0.680 (0.626–0.734)	0.815 (0.770–0.860)	0.849 (0.813–0.885)	0.665 (0.613–0.717)	0.710 (0.664–0.756)
**SA-4**	**0.615 (0.560–0.670)**	**0.704 (0.650–0.758)**	**0.830 (0.785–0.875)**	**0.862 (0.827–0.897)**	**0.688 (0.636–0.740)**	**0.731 (0.685–0.777)**

Bold indicates the best-performing model.

**Table 4:** Pairwise model comparison based on balanced accuracy. P-values were calculated from a Wilcoxon signed-rank test.

Model Comparison	*p*-Value (Histological Subtyping)	*p*-Value (Molecular Subtyping)
**SA-4 vs. DIP**	<0.001	<0.001
**SA-4 vs. MAB**	0.018	0.041
**SA-4 vs. SAB**	0.021	0.038
**SA-4 vs. SA-16**	0.025	0.048
**SA-4 vs. SA-32**	0.015	0.029
**MAB vs. DIP**	<0.001	0.009
**SAB vs. DIP**	<0.001	0.012
**MAB vs. SAB**	0.89	0.75
**SA-16 vs. MAB**	0.92	0.58

## Data Availability

The data presented in this study are openly available. The UBC-OCEAN dataset is on Kaggle at https://www.kaggle.com/competitions/ubc-ocean (accessed on 25 July 2025). The TCGA-OV dataset is available through the GDC Data Portal at https://portal.gdc.cancer.gov/ (accessed on 25 July 2025). A file mapping TCGA slide IDs to their consensus molecular subtype labels is available in the project’s GitHub repository.

## Abbreviations

The following abbreviations are used in this manuscript:

AI	Artificial Intelligence
AUC-PR	Area Under the Precision-Recall Curve
AUC-ROC	Area Under the Receiver Operating Characteristic Curve
CCC	Clear Cell Carcinoma
CI	Confidence Interval
DIP	Dynamic Instance Pruning
EC	Endometrioid Carcinoma
HGSC	High-Grade Serous Carcinoma
LGSC	Low-Grade Serous Carcinoma
MABs	Multiple Attention Branches
MC	Mucinous Carcinoma
MIL	Multiple Instance Learning
OTTA	Ovarian Tumor Tissue Analysis
SAB	Single Attention Branch
SA-4	Stochastic Attention with k=4
SA-16	Stochastic Attention with k=16
SA-32	Stochastic Attention with k=32
TCGA	The Cancer Genome Atlas
WSI	Whole-Slide Image

## Appendix A. Algorithmic Descriptions of MIL Models

This appendix provides detailed pseudocode for the forward pass of the main MIL model architectures evaluated in this study.

**Algorithm A1** Forward pass for single attention branch (SAB) model	
**Require:** Bag of instance features $H_{bag}=\left\{h_{1},\ldots ,h_{N}\right\}$, where $h_{i}\in R^{L}$	
1: $H_{transformed}\leftarrow \mathrm{FeatureExtractor}\left(H_{bag}\right)$	▷Apply FC layer + ReLU
2: $A_{scores}\leftarrow \mathrm{AttentionNet}\left(H_{transformed}\right)$	▷ Gated attention, results in N×1 scores
3: $A_{raw}\leftarrow \mathrm{Transpose}\left(A_{scores}\right)$	▷ Transpose to 1×N
4: $A_{softmax}\leftarrow \mathrm{Softmax}\left(A_{raw}\right)$	▷ Normalize scores to get attention weights
5: $M\leftarrow A_{softmax}\;\cdot \;H_{transformed}$	▷ Compute weighted average, slide representation
6: ${\hat{y}}_{logits}\leftarrow \mathrm{Classifier}\left(M\right)$	▷ Final classification
7: **return** ${\hat{y}}_{logits},A_{raw}$	

**Algorithm A2** Forward pass for multiple attention branch (MAB) model	
**Require:** Bag of instance features $H_{bag}=\left\{h_{1},\ldots ,h_{N}\right\}$, number of classes *C*	
1: $H_{transformed}\leftarrow \mathrm{FeatureExtractor}\left(H_{bag}\right)$	
2: $A_{scores}\leftarrow \mathrm{MultiBranchAttentionNet}\left(H_{transformed}\right)$	▷ Results in N×C scores
3: $A_{raw}\leftarrow \mathrm{Transpose}\left(A_{scores}\right)$	▷ Transpose to C×N
4: $A_{softmax}\leftarrow \mathrm{Softmax}\left(A_{raw},\dim=1\right)$	▷ Normalize each branch independently
5: $M\leftarrow A_{softmax}\;\cdot \;H_{transformed}$	▷ Compute *C* slide representations
6: ${\hat{y}}_{logits}\leftarrow \mathrm{empty}\;\mathrm{tensor}\;\mathrm{of}\;\mathrm{size}\;\left(1,C\right)$	
7: **for** c=1,…,C **do**	
8: ${\hat{y}}_{logits}\left[c\right]\leftarrow {\mathrm{Classifier}}_{c}\left(M\left[c\right]\right)$	▷ Apply class-specific classifier
9: **end for**	
10: **return** ${\hat{y}}_{logits},A_{raw}$	

**Algorithm A3** Forward pass for dynamic instance pruning (DIP) + SAB model	
**Require:** Bag of instance features $H_{bag}=\left\{h_{1},\ldots ,h_{N}\right\}$, pruning threshold *K*	
1: $S_{scores}\leftarrow \mathrm{ScoringNet}\left(H_{bag}\right)$	▷ Get relevance score for each instance
2: $TopK_{indices}\leftarrow \mathrm{TopK}\left(S_{scores},K\right)$
3: $H_{pruned}\leftarrow \mathrm{Select}\left(H_{bag},TopK_{indices}\right)$	▷ Select top-K instances
4: ${\hat{y}}_{logits},A_{raw\_pruned}\leftarrow \mathrm{SAB}\_\mathrm{ForwardPass}\left(H_{pruned}\right)$	▷ Process pruned bag with SAB
5: **return ** ${\hat{y}}_{logits},A_{raw\_pruned}$	
